# Impact of social support on demoralization in patients with gynecological cancer: The chain mediating role of resourcefulness and resilience

**DOI:** 10.1016/j.apjon.2025.100815

**Published:** 2025-11-05

**Authors:** Dan Liu, Silan Yang, Mingyu Li, Bing Fu, Qinjing Yang, Fu Xiang

**Affiliations:** a Department of Nursing, The Third Xiangya Hospital of Central South University, Changsha, China; b Department of Nursing, Affiliated Hospital of Jiaxing University, The First Hospital of Jiaxing, Jiaxing, China; c Department of Gynecology, The Third Xiangya Hospital of Central South University, Changsha, China; d Department of Gynecology, The People's Hospital of Xiangxi Tujia and Miao Autonomous Prefecture, The First Affiliated Hospital of Jishou University, Jishou, China

**Keywords:** Gynecology, Neoplasms, Social support, Resourcefulness, Resilience, Demoralization

## Abstract

**Objective:**

To explore the association between social support and demoralization and how resourcefulness and resilience mediate this relationship.

**Methods:**

A convenience sampling method was used. A cross-sectional study was conducted among 343 gynecological cancer patients in three hospitals in Hunan Province, China, from October 2024 to March 2025. The Social Support Scale, Demoralization Scale, Resourcefulness Scale, and Resilience Scale were used to collect data. Data analysis was conducted via descriptive statistical analysis, Spearman correlation analysis, and regression analysis to estimate direct and indirect effects via bootstrap analysis.

**Results:**

Social support was positively correlated with resourcefulness (*r* ​= ​0.395, *P* ​< ​0.001) and resilience (*r* ​= ​−0.372, *P* ​< ​0.001). Additionally, social support was significantly negatively correlated with demoralization (*r* ​= ​−0.325, *P* ​< ​0.001). Social support indirectly affected demoralization through three mediating pathways: resourcefulness (effect ​= ​−0.020; standard error ​= ​0.022; 95% confidence interval [CI] [-0.064, −0.024]), resilience (effect ​= ​−0.037; standard error ​= ​0.015; 95% CI [-0.072, -0.013]), and the resourcefulness–resilience pathway (effect ​= ​−0.038; standard error ​= ​0.013; 95% CI [-0.070, −0.018]). The indirect effect accounted for 18.35% of the total effect.

**Conclusions:**

We proved that resourcefulness and resilience mediated some of the effects of social support on demoralization. The findings of this study have implications for interventions to promote the social support of gynecological cancer patients and, in particular, to enhance resourcefulness and resilience.

## Introduction

Gynecological cancers, including cervical, endometrial cancer, and ovarian cancer, are among the most common types of cancer affecting women globally.^1^ In 2022, cervical cancer, endometrial cancer, and ovarian cancer had new occurrences of 660,000, 420,242, and 324,398 cases, respectively.^2^ With the development of medical treatment technology, the survival time of cancer patients is gradually being extended. However, a series of treatment-related outcomes makes it difficult for patients to cope, resulting in negative emotions.^3^ Demoralization is a cluster of psychological symptoms that last more than 2 weeks, and it involves an individual's subjective perceptions of an inability to cope with or a loss of control in the face of prolonged stress or illness.^4^ It is characterized by a persistent inability to cope, as well as helplessness, hopelessness, and despair.^5^ Previous studies have reported that 39.1–52.0% of cancer patients who receive a clinically significant diagnosis experience demoralization.^5^^,^^6^ Approximately 23.7–88.8% of cancer patients have different degrees of demoralization.^7^ Studies have shown that demoralization can affect the quality of life and suicidal behavior of cancer patients.^5^^,^^8^

Social support is an important factor that affects the mental health of patients with gynecological cancer,^9^ which includes subjective support, objective support (from family, peers, or the community), and support utilization.^10^ Previous studies have demonstrated that social support is negatively correlated with demoralization in gynecological cancer patients.^11^ In addition, it is significantly positively correlated with resourcefulness^12^ and resilience^13^^,^^14^ in gynecological cancer patients. In fact, resourcefulness plays a key role in managing stress and dealing with anxiety or depression.^15^ It includes personal resourcefulness and social resourcefulness and is a repertoire of cognitive behavioral self-help skills that enhance one's ability to cope with adversity.^15^ There was a negative relationship between resourcefulness and demoralization.^12^ Studies have indicated that higher levels of resourcefulness can prevent and reduce suicidal ideation in patients, alleviate demoralization.^12^^,^^16^ In addition, research has shown that resourcefulness and resilience are positively correlated.^17^ However, to the best of our knowledge, few studies have reported on the resourcefulness of patients with gynecological cancer.

As a positive psychological factor, resilience refers to a set of capacities or processes that enable individuals to successfully cope with adversities and maintain their psychological and physical well-being.^18^ Resilience and resourcefulness are two key factors that affect demoralization in patients with cancer. However, the relationship between resilience and resourcefulness in gynecological cancer patients remains unclear. Szanton and Gill posit that resilience manifests as three distinct trajectories (resistance, recovery, and rebound).^19^ While some aspects of resilience appear to be innate, others can be taught by targeting problem-solving skills, social support and coping skills.^19^^,^^20^ Resourcefulness refers to a collection of cognitive and behavioral skills that are applied creatively to handle problems and situations.^21^ Therefore, resourcefulness may influence resilience. In addition, resilience has a positive effect on the loss of demoralization.^22^^,^^23^ Previous studies have shown that social support, resourcefulness, and resilience are associated with the demoralization in cancer patients.^12^^,^^17^ Although social support has been proven to affect demoralization,^24^ the path through which resourcefulness and resilience play a role remains unclear. Clarifying the relationships between them can provide a theoretical basis for formulating targeted psychological intervention strategies, further reducing the level of hopelessness among gynecological cancer patients.

**Fig. 1:**
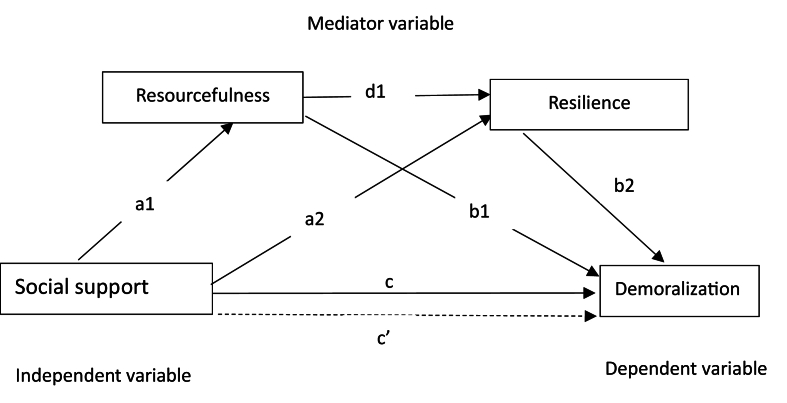
**Hypothesized serial multiple mediating model.** a1: direct effect of social support on resourcefulness; a2: direct effect of social support on resilience; b1: direct effect of resourcefulness on demoralization; b2: direct effect of resilience on demoralization; d1: direct effect of resourcefulness on resilience; c: total effect of social support on demoralization; c’: direct effect of social support on demoralization. ∗∗*P* ​< ​0.001.

In summary, we propose a model of the impact of social support on demoralization that includes resourcefulness and resilience as potential mediators on the basis of Kumpfer's resilience framework.^25^ This framework demonstrates that social support,^25^ as part of the external environmental context, has a significant effect on individual resilience. The internal characteristics of problem-solving ability are key factors affecting individual resilience, and internal and external environments act together to affect elasticity, thus effectively coping with psychological stress. We hypothesize that resourcefulness (problem-solving ability) and resilience are two mediators of the relationship between social support (external environmental context factor) and demoralization (stress reaction) after controlling for context variables (e.g., demographics) according to this model. We aim to explore the association between social support and demoralization among gynecological cancer patients and how resourcefulness and resilience mediate this association. The hypotheses are as follows: (1) social support is negatively related to demoralization, and social support is positively related to resourcefulness and resilience; (2) resourcefulness and resilience are negatively related to demoralization; and (3) resourcefulness mediates the relationship between social support and demoralization, and resourcefulness mediates the relationship between social support and demoralization via resilience. A model of the theorized hypotheses is shown in Fig. 1.

## Methods

### Study design and participants

A cross-sectional survey was conducted in 3 tertiary comprehensive hospitals in Hunan Province between October 2024 and March 2025. Convenience sampling was used to recruit patients with gynecological cancer. The inclusion criteria for participants were as follows: (1) were pathologically diagnosed with cervical cancer, ovarian cancer, endometrial cancer, etc.; (2) were ≥ 18 years old; (3) had a clear ability to express and understand the Chinese language; and (4) were able to use a smartphone. The exclusion criteria were as follows: (1) other malignant tumors or severe diseases and (2) severe cognitive impairments or mental illnesses.

**Fig. 2:**
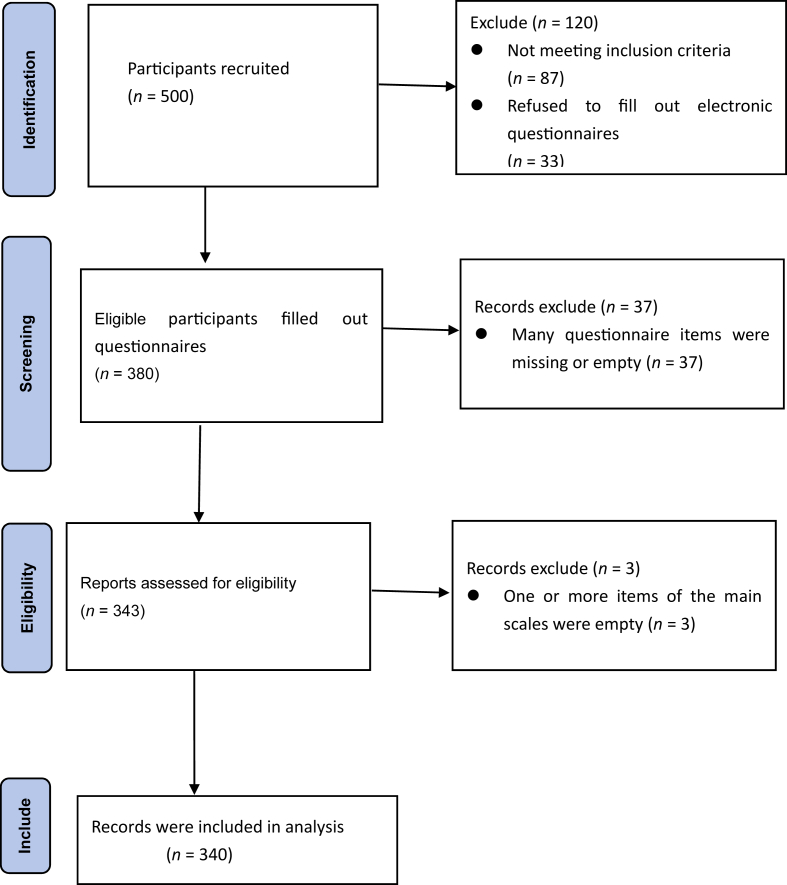
Study recruitment flowchart.

The sample size was calculated for structural equation modeling;^26^ the sample size is 10–15 times the number of observed variables, and there were 18 observed variables in this study. Hence, the initial sample size ranged from 180 to 270 patients. Considering an invalid questionnaire rate of 20% and to minimize error and enhance the credibility of the model, we ultimately determined that 324 patients were necessary. Ultimately, we recruited 343 patients for the study. Many questionnaire items were missing or empty. After the exclusion of invalid questionnaires (one or more items of the main scales were empty), only 340 questionnaires were included in the analysis (Fig. 2).

### Measures

#### General information questionnaire

Demographic information was collected by a general information questionnaire designed for the research group according to the research purpose and a rigorous literature review. It included questions on age, educational level, occupation, work status, clinical stage, therapy method and other relevant factors.

#### Social Support Rating Scale (SSRS)

Social support was investigated by the *SSRS*, which was developed by Xiao Shuiyuan in China.^10^ The scale includes 3 dimensions and 10 items, including subjective support (4 items), objective support (3 items), and support utilization (3 items). The total score was the sum of the 10 items, and the higher the score was, the more social support the patient received. The total score was 66, where a total score < 22 indicated low social support, 22 to 44 points indicated medium social support, and 45 to 66 points indicated high social support. Cronbach's α was 0.788 in this study.

#### Resourcefulness Scale (RS)

This scale was developed by Zauszniewski et al.^27^ and adapted for Chinese patients by Ke Xi et al.^28^ The scale includes two dimensions: personal resourcefulness and social resourcefulness. It includes 28 items. Each item is rated on a Likert scale ranging from 0 (not very much like) to 5 (very much like), and the total score is 140. A higher total score indicates a higher level of resourcefulness in the subject. Cronbach's α is 0.850 was this study.

#### Connor-Davidson Resilience Scale (CD-RISC)

This scale was developed by Connor et al.^29^ and translated and localized into Chinese by Yu et al.^30^ It consists of three dimensions: optimism (4 items), resilience (13 items), and self-reliance (8 items). Each item is rated on a 5-point Likert scale ranging from 0 to 4. The total score is 100, with higher scores indicating better resilience. Cronbach's α was 0.932 in this study.

#### Demoralization Scale II (DS-II)

This scale was developed by Robinson et al., in 2016.^31^ It was translated and adapted into Chinese by Ou Na and applied to cancer patients.^32^ The scale consists of 16 items across two dimensions (meaning and purpose, distress and coping ability). Each item is rated on a 3-point Likert scale. The total score is 32, with a score ≤ 9 indicating mild demoralization, 10–19 indicating moderate demoralization, and ≥ 20 indicating severe demoralization. Higher scores indicate greater levels of demoralization in patients. Cronbach's *α* for the scale was 0.896 in this study.

### Data collection

The participants and investigators were recruited through a research collaboration team at the hospital. Investigators received standardized training (uniform instructions and questionnaire item explanations). The questionnaires were input into “Wenjuan Xing” (https://www.wjx.cn/), an electronic questionnaire platform. During the patients' inpatient stays, the investigators sent a web page of the questionnaire to the participants’ mobile phones using a social media app (WeChat).^33^ Informed consent was also provided on the homepage of the questionnaire, and the subjects clicked “informed consent” before entering the response interface. All information was available only to the research members, and the exported data were encrypted and saved. For questionnaire quality control, (a) the questionnaire options were checked before submission, and (b) each IP address was allowed to complete the questionnaire only once.

### Statistical analysis

SPSS 26.0 was used for statistical analysis. Categorical data were described via frequencies and percentages, and continuous data that were not normally distributed were expressed as medians (interquartile ranges) *[M (P25, P75)]*. Spearman's correlation analysis was used to explore the correlations between the variables. These steps of the mediation analysis are the same as those in our previously published study.^34^ We used 10,000 bootstrap resamples to calculate the 95% confidence interval (CI). If the interval did not include zero, the effect was considered to be statistically significant at *P* ​< ​0.05.^34^

## Results

### Common method bias test

Harman's single-factor test was used to assess common method bias. The results revealed that nine factors had eigenvalues > 1, with the first factor explaining 23.31% of the variance, which is less than the critical standard of 40%.^35^ Therefore, there was no significant common method bias in the data.

### Characteristics of the participants and differences in demoralization, social support, resourcefulness and resilience

As shown in Table 1, a total of 340 participants with gynecological cancer were included in this study. The participants’ ages ranged from 20 to 93 years (mean ​= ​50.18, SD ​= ​11.61). The highest proportion of participants with an educational level at or below junior school was 75.88%. Most participants were married (89.41%), and 61.76% of the participants were unemployed. The average monthly household income was < 3000 CNY (68.53%). A total of 48.53% of the participants lived in rural areas. Disease diagnosis times of less than 1 year accounted for 76.47%. The percentages of mild and moderate demoralization in patients with gynecological tumors were 85% and 15%, respectively. The differences in demoralization among the participants were significant (*K* ​= ​18.854, *P* ​< ​0.001) (Table 1).

**Table 1:** Participant characteristics and differences in demoralization, social support, resilience and resourcefulness.

Variable	*n* (%2)	Demoralization [M (P_25_, P_75_)]	*K/Z*	*P*	Social support [M (SD)]	*t / F*	*P*	Resilience [M (SD)]	*t / F*	*P*	Resourcefulness [M (SD)]	*t/F*	*P*
**Age (years)**													
≤ 44	102 (30.00)	1.00 (0.00, 6.00)	*K =* 1.421	0.491	43.40 (6.34)	*F =* 3.420	0.034∗	64.11 (15.04)	*F =* 0.753	0.472	95.97 (14.68)	*F =* 1.568	0.210
45-59	184 (54.12)	1.00 (0.00, 2.50)			43.35 (5.67)			66.55 (16.54)			94.45 (17.42)		
≥ 60	54 (15.88)	1.00 (0.00, 4.00)			41.06 (6.25)			65.94 (17.00)			90.94 (19.92)		
**Educational level**													
Junior school and below Junior school	258 (75.88)	1.00 (0.00, 3.00)	*K =* 2.985	0.225	42.62 (5.82)	*F =* 2.245	0.108	65.32 (16.37)	*F =* 0.346	0.708	93.70 (17.65)	*F =* 0.820	0.441
High school	34 (10.00)	1.00 (0.00, 12.00)			44.35 (5.92)			66.62 (16.82)			96.97 (14.54)		
College	48 (14.12)	1.00 (0.00, 7.50)			44.13 (6.89)			67.25 (14.74)			95.98 (14.34)		
**Marriage**													
Unmarried	11 (3.24)	2.00 (0.50, 6.50)	*K =* 3.466	0.325	37.27 (6.25)	*F =* 18.418	0.000∗∗	66.91 (14.12)	*F =* 0.983	0.401	95.82 (17.09)	*F =* 0.655	0.580
Married	304 (89.41)	1.00 (0.00, 3.50)			43.78 (5.50)			65.77 (16.13)			94.19 (16.79)		
Divorced	12 (3.53)	3.00 (0.50, 6.50)			35.83 (5.78)			59.00 (18.82)			90.92 (21.16)		
Widowed	13 (3.82)	0.00 (0.00, 3.00)			36.38 (6.98)			69.77 (16.11)			99.85 (16.55)		
**Work status**													
Unemployed	210 (61.76)	1.00 (0.00, 4.00)	*K =* 5.266	0.153	42.33 (6.00)	*F =* 3.711	0.012∗	64.18 (17.22)	*F =* 3.342	0.019∗	92.56 (18.82)	*F =* 2.354	0.072
Employed	43 (12.65)	1.00 (0.00, 6.50)			45.37 (5.79)			69.12 (14.44)			98.00 (14.49)		
Retired	16 (4.71)	0.50 (0.00, 7.50)			44.88 (5.16)			75.63 (15.09)			94.56 (12.86)		
Others	71 (20.88)	0.50 (0.00, 2.00)			43.13 (5.99)			65.99 (13.05)			97.59 (11.87)		
**Annual income surplus (CNY)**													
< 3000	233 (68.53)	1.00 (0.00, 4.00)	*K =* 2.714	0.257	42.13 (6.06)	*F =* 9.694	0.000∗∗	64.54 (17.15)	*F =* 2.196	0.113	93.19 (18.31)	*F =* 1.793	0.168
3000-5000	61 (17.94)	1.00 (0.00, 7.00)			44.07 (5.47)			67.41 (14.97)			96.41 (14.27)		
> 5000	46 (13.53)	1.00 (0.00, 2.00)			46.02 (5.33)			69.46 (11.38)			97.48 (11.68)		
**Residential type**													
City	79 (23.24)	1.00 (0.00, 3.00)	*K =* 1.315	0.518	44.62 (6.55)	*F =* 3.836	0.023∗	66.49 (12.72)	*F =* 1.385	0.252	96.70 (14.54)	*F =* 2.658	0.072
Town	96 (28.24)	1.00 (0.00, 4.50)			42.36 (5.68)			67.57 (14.20)			96.13 (13.03)		
Rural area	165 (48.53)	1.00 (0.00, 4.00)			42.60 (5.82)			64.27 (18.50)			92.19 (19.61)		
**Family members diagnosed with gynecological cancer**													
Yes	11 (0.29)	2.00 (0.00, 7.00)	*Z = -*0.771	0.441	43.64 (4.93)	*t* = 0.126	0.723	64.73 (12.34)	*t* = 0.043	0.836	97.91 (13.03)	*t* = 0.500	0.479
No	329 (96.76)	1.00 (0.00, 4.00)			42.98 (6.05)			65.75 (16.30)			94.23 (17.05)		
**Times of hospitalizations**													
1	162 (47.65)	1.00 (0.00, 3.00)	*K =* 5.642	0.130	42.97 (5.91)	*F* = 0.747	0.524	65.59 (17.56)	*F* = 0.341	0 .796	95.06 (18.64)	*F* = 0.276	0.843
2-3	155 (45.59)	1.00 (0.00, 3.50)			43.17 (6.03)			65.94 (14.41)			93.95 (15.49)		
4-5	13 (3.82)	2.00 (0.00, 11.00)			43.54 (7.92)			68.08 (16.63)			92.08 (10.00)		
>5	10 (2.94)	1.50 (1.00, 12.00)			40.30 (4.57)			61.40 (19.50)			91.80 (17.81)		
**Primary caregiver**													
Husband	181 (53.24)	1.00 (0.00, 4.00)	*K =* 3.221	0.522	44.17 (5.57)	*F =* 4.725	0.001∗∗	64.72 (14.69)	*F =* 0.657	0.622	94.41 (13.75)	*F =* 2.110	0.079∗
Parents	18 (5.29)	2.00 (1.00, 8.00)			39.50 (6.28)			65.94 (14.48)			98.78 (19.63)		
Son/Daughter	99 (29.12)	1.00 (0.00, 3.00)			41.96 (6.30)			67.49 (18.72)			93.45 (21.13)		
Brothers/Sisters	27 (7.94)	1.00 (0.00, 5.00)			42.56 (6.08)			67.37 (16.72)			99.37 (15.80)		
Others	15 (4.41)	1.00 (0.00, 2.00)			40.80 (5.85)			62.87 (16.72)			85.13 (16.78)		
**Length of disease diagnosis**													
<1 year	260 (76.47)	1.00 (1.00, 3.50)	*K =* 2.052	0.562	42.87 (6.23)	*F =* 0.420	0.739	65.60 (16.92)	*F =* 0.447	0.720	94.18 (17.57)	*F =* 0.431	0.731
1-3 years	50 (14.71)	1.00 (0.00, 3.00)			43.78 (4.68)			66.06 (14.82)			95.74 (15.70)		
4-5 years	13 (3.82)				43.54 (7.73)			70.00 (10.59)			96.69 (13.37)		
>5 years	17 (5.00)				42.35 (4.72)			63.24 (11.22)			90.94 (12.71)		
**Treatment method**													
First diagnosis without treatment initiation	100 (29.41)	1.00 (0.00, 4.00)	*K =* 1.780	0.776	42.88 (6.45)	*F =* 2.613	0.035∗	64.74 (18.12)	*F =* 2.293	0.059	92.00 (17.35)	*F =* 3.718	0.006∗
Surgical operation/Radiotherapy/Chemotherapy	224 (65.88)	1.00 (1.00, 3.50)			43.35 (5.71)			66.76 (15.14)			96.09 (16.11)		
Surgical operation+Radiotherapy+Chemotherapy	4 (1.18)	4.00 (0.00, 10.00)			42.75 (4.79)			68.75 (21.50)			68.50 (31.85)		
Surgical operation+Radiotherapy/Chemotherapy	6 (1.76)	1.00 (1.00, 4.00)			37.83 (4.96)			57.17 (8.42)			90.17 (13.86)		
Radiotherapy+Chemotherapy	6 (1.76)	2.00 (1.00, 12.00)			37.50 (7.42)			49.67 (13.31)			89.83 (16.58)		
**Clinical stage**													
Unknown	291 (85.59)	1.00 (0.00, 3.00)	*K =* 1.213	0.750	43.36 (5.82)	*F =* 2.986	0.031∗	65.98 (16.07)	*F =* 0.613	0.607	94.54 (17.15)	*F =* 0.985	0.400
I	9 (2.65)	2.00 (0.00, 12.00)			41.67 (6.93)			62.00 (12.04)			97.67 (13.12)		
II	17 (5.00)	2.00 (0.00, 10.00)			39.41 (6.24)			61.53 (20.36)			87.88 (16.84)		
III	23 (6.76)	1.00 (0.00, 2.50)			41.61 (7.11)			67.04 (15.72)			95.43 (15.22)		
**Types of caner**													
Cervical cancer	52 (15.29)	1.00 (0.00, 5.50)	*K =* 7.439	0.059	40.42 (5.95)	*F =* 5.875	0.001∗∗	64.29 (18.61)	*F =* 1.248	0.292	90.69 (20.44)	*F =* 1.089	0.354
Endometrial cancer	10 (2.94)	1.50 (1.00, 4.00)			41.90 (5.84)			64.00 (19.39)			91.80 (21.52)		
Ovarian cancer	7 (2.06)	8.00 (3.50, 11.50)			38.57 (5.74)			55.43 (5.97)			94.00 (12.82)		
Unknown	271 (79.71)	1.00 (0.00, 3.00)			43.65 (5.88)			66.32 (15.68)			95.15 (16.07)		
**Self character**													
Optimistic	145 (42.65)	1.00 (0.00, 2.00)	*K =* 18.854	0.000∗∗	43.90 (5.57)	*F =* 2.090	0.101	68.73 (16.92)	*F =* 3.562	0.015∗	96.28 (18.56)	*F =* 1.774	0.152
Easy to get angry and pursue perfection	13 (3.82)	8.00 (2.00, 12.00)			41.69 (7.80)			58.15 (12.03)			88.08 (17.28)		
Impatient and impulsive	81 (23.82)	1.00 (0.00, 6.00)			42.07 (6.64)			64.42 (15.27)			91.96 (15.15)		
Easygoing and quiet	101 (29.71)	1.00 (0.00, 2.00)			42.62 (5.75)			63.42 (15.58)			94.29 (15.52)		

*N, population size; Z, MannÂ₠“Whitney U Tes*t; *K*, KruskalÂ₠“Wallis H Tes*t; t, t* test*; F,F-test; P, P value;* ∗*P*Â <Â 0.05, ∗∗*P*Â <Â 0.001; M (P25, P75), medians (interquartile ranges); M (SD), mean (standard deviation); CI, confidence interval.

### Correlations between demoralization, social support, resourcefulness and resilience

The basic descriptive data for these variables are shown in Table 2. The distribution score of demoralization was 1.00 (0.00, 4.00) (range ​= ​34–170). The mean total score for social support was 43.00 ​± ​6.01 (range ​= ​12–66), that for resourcefulness was 94.35 ​± ​16.93 (range ​= ​0–140), and that for resilience was 65.72 ​± ​16.17 (range ​= ​0–100). Spearman's correlations revealed that social support was significantly positively correlated with resourcefulness (*r* ​= ​0.395, *P* ​< ​0.001) and resilience (*r* ​= ​−0.372, *P* ​< ​0.001). Additionally, social support was significantly negatively correlated with demoralization (*r* ​= ​−0.325, *P* ​< ​0.001).

**Table 2:** Descriptive statistics and correlation of demoralization, social support, resilience and resourcefulness (*N* ​= ​340).

Item	Range	[M (P_25_, P_75_)]/M ​± ​SD	95% CI	1	2	3	4
1 Demoralization	0–32	1.00 (0.00, 4.00)	2.64–3.63	1			
2 Social support	12–66	43.00 ​± ​6.01	42.36–43.64	−0.325∗∗	1		
3 Resilience	0–100	65.72 ​± ​16.17	64.00–67.45	−0.430∗∗	0.372∗∗	1	
4 Resourcefulness	0–140	94.35 ​± ​16.93	92.54–96.15	−0.366∗∗	0.395∗∗	0.541∗∗	1

*P, P value;* ∗∗*P* ​< ​0.001; M (P25, P75), medians (interquartile ranges); M ​± ​SD, mean ​± ​standard deviation; CI, confidence interval.

### Chain-mediating role of resourcefulness and resilience in the relationship between social support and demoralization

**Fig. 3:**
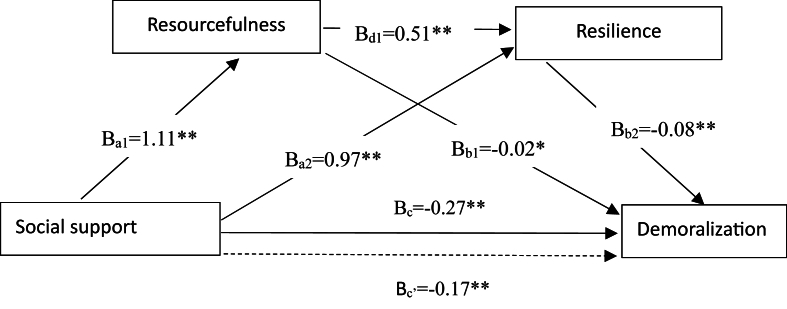
Serial multiple mediating model with resourcefulness and resilience as mediators in the relationship between social support and demoralization.

The data in Table 3 show that all the individual paths between the key variables in the model were significant after the covariates were accounted for. Greater social support was associated with greater resourcefulness (*β* ​= ​1. 11, 95% CI: 0.780, 1.413) and resilience (*β* ​= ​0.97, 95% CI: 0.685, 1.256). Both higher resourcefulness and resilience were also associated with lower demoralization (*β* ​= ​−0.02, 95% CI: −0.059, −0.023; *β* ​= ​−0.08, 95% CI: −0.117, −0.054, respectively). Additionally, the total effect (Bc) and the total direct effect (Bc’) of social support on demoralization were found to be significant (B_c_ ​= ​−0.27, SE ​= ​0.05, *t* ​= ​−5.74, *P* ​< ​0.001; B_c’_ ​= ​−0.17, SE ​= ​0.05, *t* ​= ​−3.41, *P* ​< ​0.001) (Fig. 3).

**Table 3:** Results of the regression analyses testing the serial multiple mediation effect of resilience and resourcefulness in the relationship social support and demoralization (*N* ​= ​340).

Predictors	Direct effect (SE)			Total effect (SE)
	Resourcefulness	Resilience	Demoralization	Demoralization
Constant	47.50 (7.76)∗∗	27.06 (6.91) ∗∗	17.63 (2.38) ∗∗	14.71 (2.19) ∗∗
Social support	1.11 (0.16)∗∗	0.97 (0.14) ∗∗	−0.17 (0.05) ∗∗	−0.27 (0.05) ∗∗
Resourcefulness		0.51 (0.07) ∗∗	−0.02 (0.02) ∗	
Resilience			−0.08 (0.02) ∗∗	
Self character	−0.30 (0.69)	−1.28 (0.66)	−0.13 (0.17)	−0.03 (0.18)
*R*^2^	0.40∗∗	0.39∗∗	0.44∗∗	0.34∗∗

*P, P value;* ∗∗*P* ​< ​0.001; SE, standard error; Unstandardized regression coefficients (beta) with standard error in parentheses are presented.

In accordance with the findings above, three paths were used to determine the effect of social support on demoralization, as shown in Table 4. There was a significant sequential indirect effect of social support on demoralization through resourcefulness and resilience [path a1d1b2: effect ​= ​−0.038, SE ​= ​0.013; 95% CI (−0.070, −0.018)]. Additionally, simple mediation paths from social support to demoralization via resourcefulness [path a1b1: Effect ​= ​−0.020, SE ​= ​0.022; 95% CI (−0.064, −0.024)] and from social support to demoralization through resilience [path a2b2: Effect ​= ​−0.037, SE ​= ​0.015; 95% CI (−0.072, −0.013)] were found. The separate mediating effects and the serial-multiple mediation between the three models were not at a zero-point estimate interval within the 95% CI, and resourcefulness and resilience had significant mediating effects on demoralization. The direct effect of social support on demoralization was −0.173, accounting for only 64.80% of the total effect (−0.267). Second, the total indirect effect of social support on demoralization through resourcefulness and resilience was −0.049, which accounted for 18.35% of the total effect (−0.267).

**Table 4:** Bootstrapped point estimates with standard errors and 95% CIs for all indirect effects between social support and demoralization (*N* ​= ​340).

	(Path)	Effect	SE	Bootstrapping 95% CI	
				Lower	Upper
Direct effects	(c’)	−0.173	0.051	−0.273	−0.735
Indirect effects					
Via resourcefulness	(a1b1)	−0.020	0.022	−0.064	−0.024
Via resilience	(a2b2)	−0.037	0.015	−0.072	−0.013
Via resourcefulness and resilience	(a1d1b2)	−0.038	0.013	−0.070	−0.018
Total indirect effect		−0.049	0.020	−0.134	−0.055
Total effect		−0.267	0.047	−0.359	−0.176

*c’,* direct effect of social support on demoralization; a1, direct effect of social support on resourcefulness; a2, direct effect of social support on resilience; b1, direct effect of resourcefulness on demoralization; b2, direct effect of resilience on demoralization; d1, direct effect of resourcefulness on resilience; CI, confidence interval; SE, standard error; If the CI does not include zero, the effect is statistically significant at *P* ​< ​0.05.

## Discussion

The results of this study support the initial research hypotheses after controlling for covariate variables: (1) social support, resourcefulness and resilience are significantly associated with each other and negatively related to demoralization; and (2) resourcefulness and resilience chain mediate the relationship between social support and demoralization.

Our study revealed that the social support of participants was positively correlated with resourcefulness and resilience, which is consistent with the findings of previous research.^12^^,^^14^ Social support includes assistance from friends, neighbors, families, colleagues and community organizations etc., When social support is sufficient, individuals can seek external support and resources when needed, making full use of available social resources such as community support.^15^ The resourcefulness of individuals could be high. Similarly, individuals who tend to actively seek help from others, actively utilize social resources, and develop effective coping skills have a higher level of resilience.^36^

In addition, social support was significantly negatively related to demoralization, which is consistent with the findings of previous studies.^11^^,^^13^^,^^24^ Social support includes the tangible and psychological assistance that individuals receive from family, peers, or the community during stressful situations.^37^ Social support has been identified as a significant source of comfort, aiding patients in navigating the challenges posed by their condition.^24^ Social support can directly influence patients' attitudes and behaviors toward coping with illness.^38^ Patients with higher levels of social support are more likely to feel that they are important in the minds of others, enabling them to receive greater assistance during the treatment process. This fosters more positive attitudes among patients toward emotions, mental health, and other aspects, thus resulting in lower levels of demoralization.

This study revealed that resourcefulness mediates the relationship between social support and demoralization. These results are similar to those of Yingtao Meng's research.^12^ Resourcefulness emphasizes that individuals can continuously accumulate beliefs and cognitive behavioral skills through learning and education in life to cope with stress.^39^ Individuals can use these beliefs and coping skills to control and adjust the impact of internal and external stressors, helping them adapt to their own physical and mental health conditions.^21^ Higher levels of resourcefulness in gynecological cancer patients mean that they can better utilize the beliefs and coping skills that they have acquired, as well as available social resources, to eliminate the burden of self-perception, better manage stress, and thus reduce the level of demoralization.

Resilience partly mediates the relationship between social support and demoralization. When social support levels increase, patients' resilience strengthens, and their demoralization is alleviated. Our findings are similar to those of a study reported by Scandurra C et al., who reported that as resilience increased, demoralization decreased.^40^ In addition, social support provides the necessary information and resources and promotes positive coping strategies. Kumpfer's resilience framework also highlights families, communities and other social support resources as protective factors that perform a buffering function during crisis incidents.^25^ Therefore, patients with higher levels of social support exhibit greater resilience. As a positive psychological factor, resilience helps individuals cope with various adverse situations and face adversity. Gynecological cancer patients with higher resilience could adapt to utilize various resources to alleviate negative emotions, regulate their own condition, and confront the disease with an optimistic mindset, thus resulting in lower levels of demoralization. These findings suggest that health care professionals should pay attention to patients' resilience levels, mobilize favorable resources, assist patients in adjusting their psychological state, reduce negative emotions, and enhance both physical and psychological comfort, thus lowering the incidence of demoralization.

This study revealed that resourcefulness and resilience chain mediate the relationship between social support and demoralization. These new findings are consistent with our hypothesis that resourcefulness mediates the relationship between social support and demoralization via resilience. Social support is important for alleviating demoralization and enhancing resourcefulness and resilience, which in turn promotes improvements in gynecological cancer patients' demoralization. Previous research has indicated that social support serves as a crucial protective factor promoting the resourcefulness of cancer patients. High-quality social resources can enhance individuals' ability to activate coping skills in adverse situations, thus promoting an improvement in their resourcefulness level.^12^ In addition, resourcefulness includes personal resourcefulness, which refers to the continuous accumulation of an individual's knowledge and experience through events, imitation, experience, education, and other means during the individual's growth process. This forms beliefs, cognitions, and behavioral skills for coping with stress.^15^^,^^21^ Personal resourcefulness is essentially a combination of cognition and behavior. The resilience framework demonstrates that cognitive and behavioral factors affect an individual's internal resilience.^25^ Therefore, the higher the level of resourcefulness in gynecological cancer patients is, the greater their resilience, and the lower the patients' demoralization level. Therefore, medical staff should pay attention to the role of resourcefulness and resilience in the levels of disorientation among patients with gynecological cancer and help these patients improve their psychological state by identifying, intervening, and establishing support networks.

### Limitations and future directions

While this study yielded significant findings, the following limitations still exist: (1) The study was conducted in a single region and utilized convenience sampling. The sample may exhibit central tendencies, which could impact the generalizability of the results. Future researchers should consider a multiregional design and employ random sampling methods to increase the representativeness of the sample. (2) The study was a cross-sectional survey and could not examine the dynamic changes in relationships between these variables. Future studies should use longitudinal designs to reveal the causal relationships and trends between these variables at different time points.

### Implications for clinical care

This study has important theoretical and practical significance for addressing demoralization in patients with gynecological cancer, and it provides valuable information for developing effective intervention strategies. Clarifying the psychological pathway of gynecological cancer patients is crucial for addressing their demoralization by enhancing resourcefulness and resilience. This study expands the potential factors affecting demoralization in gynecological cancer patients and reveals effective pathways to alleviate demoralization. Therefore, clinical and medical staff should assist patients in fully utilizing social support resources, provide interventions to enhance their resourcefulness and resilience, and alleviate their demoralization.

## Conclusions

The findings of this study suggest that social support is positively correlated with resourcefulness and resilience among gynecological cancer patients and negatively correlated with demoralization. Notably, social support has a significant direct effect on demoralization, while resourcefulness and resilience partially mediate the relationship between social support and demoralization. Furthermore, they serve as chain mediators in this relationship. Based on the pathway model, targeted intervention strategies such as rapport building, stress management, goal setting, cognitive reframing, meaning-making, and family integration may be developed to increase patients' resourcefulness and resilience,^41^ thus addressing their demoralization and promoting their psychological rehabilitation.

## CRediT authorship contribution statement

Dan Liu: Conceptualization, Methodology, Funding acquisition, Formal analysis, Writing - original draft, Writing - review and editing, Project administration. Silan Yang: Conceptualization, Methodology, Funding acquisition, Data curation, Writing - original draft, Writing - review and editing, Supervision. Mingyu Li: Resources, Validation, Data curation. Bing Fu: Data curation, Supervision. Qinjing Yang: Data curation, Supervision. Fu Xiang: Writing - review and editing, Data curation. All authors have read and approved the final manuscript.

## Ethics statement

This study was approved by the Medical Ethics Committee of The People's Hospital of Xiangxi Tujia and Miao Autonomous Prefecture (The First Affiliated Hospital of Jishou University) (Approval No. EC-LCKY2024047) and was conducted in accordance with the 1964 Helsinki Declaration and its later amendments or comparable ethical standards. For the two sites without independent ethics committees, oversight was provided by the above committee with support from local study departments. All participants provided written informed consent.

## Data availability statement

The data that support the findings of this study are available from the corresponding author, SY, upon reasonable request. The data are not publicly available due to their containing information that could compromise the privacy of our participants.

## Declaration of generative AI and AI-assisted technologies in the writing process

No AI tools/services were used during the preparation of this work.

## Funding

This study was supported by the Hunan Provincial Youth Natural Science Foundation Project (Grant No. 2024JJ6611) and 10.13039/501100017594Medical Science and Technology Project of Zhejiang Province (Grant No. 2022KY1241). The funders had no role in considering the study design or in the collection, analysis, interpretation of data, writing of the report, or decision to submit the article for publication.

## Declaration of competing interest

The authors declare no conflict of interest.
